# Effects of Volume Fraction and Surface Area of Aggregates on the Static Yield Stress and Structural Build-Up of Fresh Concrete

**DOI:** 10.3390/ma13071551

**Published:** 2020-03-27

**Authors:** Irina Ivanova, Viktor Mechtcherine

**Affiliations:** Institute of Construction Materials, TU Dresden, 01069 Dresden, Germany

**Keywords:** fresh concrete, rheology, static yield stress, structural build-up, aggregates, packing fraction

## Abstract

With increasing interest in the use of additive manufacturing techniques in the construction industry, static rheological properties of fresh concrete have necessarily come into focus. In particular, the knowledge and control of static yield stress (SYS) and its development over time are crucial for mastering formwork-free construction, e.g., by means of layered extrusion. Furthermore, solid understanding of the influences of various concrete constituents on the initial SYS of the mixture and the structural build-up rate is required for purposeful material design. This contribution is concentrated on the effect of aggregates on these rheological parameters. The volume fraction of aggregates was varied in the range of 35% to 55% by volume under condition of constant total surface area of the particles. The total surface area per unit volume of cement paste was equal to 5.00, 7.25 and 10.00 m²/L, conditioned on the constant volume fraction of aggregates. Both variations were enabled by changing the particle size distributions of the aggregates while holding the cement paste composition constant for all concrete mixtures. To characterise the SYS and the structural build-up, constant shear rate tests with a vane-geometry rotational rheometer were performed. It was found that in the ranges under investigation the variation in volume fraction had a more pronounced effect on the static rheological properties of concrete than did the variation in surface area. An accurate mathematical description of the relationship between the initial SYS of concrete and the relative volume fraction of aggregate based on the Chateau–Ovarlez–Trung model was proposed. Challenges in deriving a similar relationship for the structural build-up rate of concrete were highlighted.

## 1. Introduction

Triggered by the speedy development of additive manufacturing technologies in the construction sector, static yield stress and structural build-up of fresh concrete come more and more into the focus of both scientists and practitioners [1,2,3]. This contrasts with previous decades, when the major interest was to understand and to shape such processes as mixing, pumping, filling formwork, and compacting, and hence, in-depth investigations in concrete rheology were predominantly dedicated to dynamic properties. The research on the static properties of concrete occurred mainly in the context of predicting formwork pressure by self-consolidating concrete [4,5,6] and of estimating gas migration in oil well cement slurries [7]. However, first attempts to upscale digital fabrication approaches have already demonstrated the urgent need to comprehend and control the behaviour of fresh concrete at rest. For instance, in the case of extrusion-based 3D-printing—due to the absence of formwork—the freshly deposited layers must retain their shape under their own weight and the weight of the subsequently placed layers [8,9]. Time intervals between deposition of subsequent layers should as well be relatively short to ensure sufficient adhesion between layers, thus avoiding of cold joints on one hand [2,10], and enabling a sufficiently high rate of construction and cost efficiency of the new technology on the other. Obviously, the static rheological properties and their development over time must comply with the specific requirements of particular technologies or even individual applications. Thus, it is necessary to gain a solid understanding of the effects of various concrete constituents on the mixture’s initial static yield stress (SYS) and the structural build-up rate, i.e., structuration rate which characterises the increase in static yield stress due to flocculation and early hydration.

This research concentrates on the role of aggregates with respect to static rheological properties. The content of aggregates is usually very high in concrete; actually, it is, in most cases, the major constituent. The content, particle size distribution, particle shapes, and surface morphology as well as other properties of aggregates can significantly affect the behaviour of fresh mixtures [11,12,13]. Numerous attempts were made to develop analytical models for optimal particle packing of aggregates, including the well-known equations by Fuller and Thompson [14], Andreasen and Andersen [15], Funk and Dinger [16]. Many investigations have been also dedicated to establishing a link between the dynamic rheological properties of a suspension and a suspending medium, starting with Einstein’s equation for low-concentration suspensions of spherical particles in a Newtonian fluid [17]. Analytical models for predicting the viscosity of concentrated suspensions were suggested by Krieger and Dougherty [18], Chong et al. [19], Quemada [20], and others. Krieger and Dougherty [18] provided an equation for non-Newtonian flow in suspensions of rigid spherical particles; the model of Chong et al. [19] is limited to polydisperse Newtonian fluid suspensions of spherical glass beads; Quemada’s equation is valid for concentrated, disperse systems of Newtonian behaviour. Three parameters for particles were featured in the known models: volume fraction φ, maximum volume fraction $\varphi_{max}$, at which the viscosity becomes infinite, and intrinsic viscosity [η] which represents the effect of the particle shape.

Although the significance of the existing models should not be underestimated, they cannot be directly applied to concrete, which is a suspension of non-spherical, polydisperse particles in a non-Newtonian, yield stress, thixotropic fluid with time-dependent properties. One model for predicting the viscosity of concrete was presented by Farris in 1968 [21]. He proposed the treating of fresh concrete as a suspension consisting of three phases, namely cement paste, sand and gravel. The modified version of his model was suggested by Choi et al. [22] and successfully used to describe the behaviour of concrete during pumping.

Less attention has been paid to the yield stress of suspensions. Chateau et al. [23] suggested a model for linking the yield stress of suspension $\tau_{0,s}$ consisting of monodisperse spherical particles and the yield stress of a non-reactive yield stress fluid $\tau_{0,f}$. This is now known as the Chateau–Ovarlez–Trung model and reads as follows: $\tau_{0,rel}=\tau_{0,s}/\;\tau_{0,f}=\sqrt{\left(1-\varphi \right)\;{\left(1-\varphi /\varphi_{max}\right)}^{-2.5\varphi_{max}}}$. Mahaut et al. [24] proved the model to be equally valid for a cement-based suspension of glass beads. However, they emphasized that instead of the maximum volume fraction, a lower value should be taken in order to make the model and the experimental results closely comparable. They argued that direct contacts among aggregate particles are established before their volume fraction reaches $\varphi_{max}$. As soon as the particles are in direct contact, not only hydrodynamic but also frictional particle interactions occur, and the model becomes inapplicable. Relying upon the Roussel’s equation for structural build-up rate [25], the authors also stated that the same model is able to describe the ratio between the structural build-up rates of suspension $A_{thix,s}$ and of cement paste $A_{thix.p}$: $A_{thix,rel}=A_{thix,s}/A_{thix.p}=\sqrt{\left(1-\varphi \right){\left(1-\varphi /\varphi_{max}\right)}^{-2.5\varphi_{max}}}$. It should be noted that the experimental data used as a basis for this conclusion were obtained for only short resting times of 2, 4 and 6 min after a strong stirring of the suspension.

Lecompte et al. [26] attempted to use the Chateau–Ovarlez–Trung model to predict the structural build-up rate of self-consolidating concrete by introducing a random loose packing fraction $\varphi_{RLP}$ instead of $\varphi_{max}$. The experimental results were collected for the period between 10 min and 40 min after completion of mixing and showed a noticeable discrepancy with the predicted values. Changing from the maximum volume fraction to the random loose packing fraction was proposed because, as described above, the latter already represents direct contacts between aggregate particles.

Perrot et al. [27] performed a similar study on cementitious mortars containing rigid fibre. While determining the structural build-up rate for resting times of 0 to 40 min, the authors avoided pre-shearing the sample before each measurement. Instead of maximum volume fraction $\varphi_{max}$, random loose packing fraction $\varphi_{RLP}=0.8\varphi_{max}$ was implemented in the Chateau–Ovarlez–Trung model. The experimental data for both relative yield stress and relative structural build-up rate showed good correspondence with the model up to a relative volume fraction $\varphi /\varphi_{RLP}$ of 0.8.

Hafid et al. [12] drew attention to the effect of the particle shape on the evolution of the relative yield stress of suspensions with increasing volume fraction of aggregates. They highlighted that a decrease in the sphericity of the particles resulted in higher relative yield stress at the same volume fraction when the volume fraction was greater than or equal to 0.30. The data were obtained using polystyrene beads and sand with various particle shapes, and a model laboratory yield stress fluid.

The abovementioned investigations have contributed notably to the ability to predict static rheological properties of concrete when the properties of the constitutive paste and the parameters of the aggregates are known. Such predictions have great value in simplifying the process of mixture design in terms of static yield stress. However, the results of these investigations might not be directly applicable for digital fabrication technologies such as layered extrusion [1,28], shotcrete 3D printing [29], and smart dynamic casting [8]. The reason is that they were obtained either for model materials or, in the cases of mortar and concrete, mostly for the pre-sheared samples with a maximum resting period of 40 min and with materials containing considerable dosages of superplasticizers. Superplasticizers can exert a retarding effect on the cementitious materials, leading to slower structural build-up [30]. Under in situ conditions, deposited layers are not subjected to any stirring; therefore, structural build-up develops faster than in pre-sheared material. In [31] the authors experimentally proved that pre-shearing of cementitious materials before the actual rheological test leads to a pronounced underestimation of the parameter $A_{thix}$. It is also worthwhile noting that the structural build-up for 3D printed concrete should be studied over a longer time than 40 min after mixing because the duration of printing often exceeds this interval. Moreover, concrete compositions used for layered extrusion have lower superplasticizer contents, so that that shape stability of printed layers can be achieved. Thus, further research on the effects of aggregates on the structural build-up of cement-based composites is required while taking into consideration the specific aspects of 3D concrete printing.

Interestingly, little attention has been dedicated to the possibility of physical-chemical interactions between cement paste and aggregate particles and, by extension, to the corresponding presumable influence on the structural build-up processes in concrete. If such influence exists, then the surface area of aggregates must be of importance. Firstly, water adsorption and wetting of aggregate surface should affect the composition of the cement paste in concrete. Larger surface areas of aggregates usually result in higher water demand; thus, the actual water-to-binder (w/b) ratio in the constitutive cement paste will be reduced to some extent, possibly leading to faster structural build-up. Secondly, the surface of aggregate particles can be considered as the interphase where predominant formation of hydration products occurs [32]. It is reasonable to assume that, since the interphase processes are generally more intensive in the case of finer particle sizes with their larger surface area, the use of finer particles will lead to some increase in structural build-up rate for the same volume fraction of aggregate in the mixture.

Taking into account these hypotheses along with the existing findings on the effect of volume fraction of aggregates on the static rheological properties of cementitious materials, the authors decided to investigate in this work: (i) the effect of the volume fraction under conditions of constant surface area of the aggregates, (ii) the effect of the surface area under conditions of the constant volume fraction of aggregates. Both scenarios could be staged by variation of the particle size distribution of the aggregates. The volume fraction of aggregates was increased from 0.35 to 0.55, in increments of 0.05. The surface areas, calculated per unit volume of cement paste, was equal to 5.00, 7.25 and 10.00 m^2^/L, while the cement paste composition was maintained constant for all mixtures under investigation.

## 2. Materials and Methods

### 2.1. Design of Concrete Mixtures

Portland cement CEM I 42.5 R (HeidelbergCement AG, Heidelberg, Germany), quartz sand 0–2 mm, sand 2–4 mm and gravel 4–8 mm (Ottendorf-Okrilla GmbH & Co.KG, Laußnitz, Germany) were used in this investigation. The sand and the gravel were natural washed river aggregates comprising 93.8% of quartz. For this research, they were treated under laboratory conditions as follows: dried until a constant weight was reached; the sand was then sieved to obtain narrow fractions of 0.125–0.250 mm, 0.25–0.50 mm, 0.5–1.0 mm and 1–2 mm. Narrow fractions were required for precise control over the particle size distributions in the concrete mixtures.

A mix design was performed under consideration of the particle shape of the aggregates using the values of the average sphericity obtained for each fraction. Sphericity was determined by means of dynamic image analysis with the particle size and shape analyser Sympatec QICPIC, which can be applied for characterization of particles ranging from below 1 µm to 34 mm and is equipped with a high-speed camera capturing particle projections with a frequency of 500 frames per second. Sphericity is defined by Wadell [33] as the ratio of the surface area of a sphere to the surface area of an actual, irregularly shaped particle of equivalent volume. The following values were obtained: 0.84 for the fraction 0.125–0.250 mm, 0.83 for the fraction 0.25–0.50 mm, 0.82 for the fraction 0.5–1.0 mm, 0.86 for the fraction 1–2 mm, 0.87 for the fraction 2–4 mm, and 0.86 for the fraction 4–8 mm.

Ratios between the aggregate fractions were varied to obtain the required value of surface area of aggregates per unit volume of cement paste. This parameter shows which aggregate surface area is exposed to the specific amount of cement paste and is defined for each separate fraction using Equation (1):

(1) $$A_{ag,i}^{V_{p}}=\frac{6m_{ag}}{\rho_{ag}\psi V_{p}d_{av}}$$

where $m_{ag}$ is the weight of the aggregate fraction in the mixture, $\rho_{ag}$ is the true density of the aggregate, ψ is the average sphericity of the aggregate particles, $V_{p}$ is the volume of the cement paste, $d_{av}$ is the average particle diameter, calculated as the mean value between the smallest diameter and the largest diameter of the particles in the given fraction. The total surface area of aggregate per unit volume of cement paste $A_{ag}^{V_{p}}$ was derived as the sum of the values calculated using Equation (1) for each separate aggregate fraction.

Grading curves, maximum volume fractions and random loose packing fractions of the aggregate compositions are provided in Table 1; for testing methods see Section 2.2. The concrete compositions as designed are given in Table 2 along with the workability of the concrete measured using the Haegermann flow table (HFT); for testing method see Section 2.3. For each set of tests, 4 L of concrete was prepared in accordance with the procedure described in [31]. In all cases, w/b of the cement paste was kept constant, equal to 0.4. In case (i), as described in the introduction, the volume fraction φ of the aggregates was varied from 0.35 to 0.55, while $A_{ag}^{V_{p}}$ was kept constant at 7.25 m^2^/L. Then for case (ii), the authors selected φ of 0.35, 0.45 and 0.55 and for each of these values tested the concrete compositions with $A_{ag}^{V_{p}}$ of 5.00, 7.25 and 10.00 m^2^/L. In all cases the required characteristics of the aggregates were achieved by varying the ratios between the individual fractions. In addition, the cement paste was tested.

**Figure 1:**
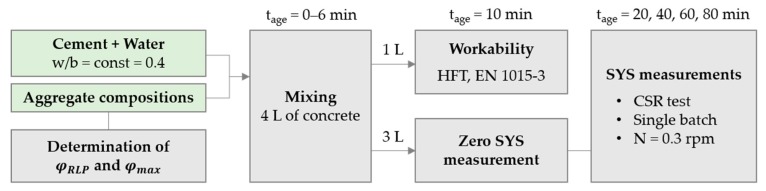
Summary of the experimental program.

The experimental program is summarized in Figure 1.

### 2.2. Determination of Packing Fraction

The values of both random loose packing fraction and maximum volume fraction were experimentally determined for each aggregate composition. In doing this, 8 kg aggregate mix was prepared and put into a steel cylinder with an inner diameter of 149 mm and a height of 300 mm using a shovel. Then the top portion was carefully levelled with a spatula, trying to apply the least possible force. The distance from the top portion of the aggregate mix to the top of the cylinder ${∆}_{RLP}$ was measured to within an accuracy of 1 mm. After that the cylinder was put on a vibration table, and a 28 kg piston was placed on the aggregates. The cylinder with aggregates and piston was vibrated at a frequency of 50 Hz for 2 min. Subsequently the piston was removed and the distance from the top surface of the aggregate to the top of the cylinder ${∆}_{max}$ was recorded.

Random loose packing fraction $\varphi_{RLP}$ and maximum volume fraction $\varphi_{max}$ were calculated using Equations (2) and (3), respectively [34]:

(2) $$\varphi_{RLP}=\frac{4m_{ag}}{\rho_{ag}\pi d_{cyl}^{2}\left(h_{cyl}-{∆}_{RLP}\right)}$$

(3) $$\varphi_{max}=\frac{4m_{ag}}{\rho_{ag}\pi d_{cyl}^{2}\left(h_{cyl}-{∆}_{max}\right)}$$

where $m_{ag}$ is the weight of the aggregate, $\rho_{ag}$ is the true density of the aggregate, $d_{cyl}$ and $h_{cyl}$ are the diameter and the height of the steel cylinder, respectively.

Each composition was tested three times, or until the difference between the obtained values was less than or equal to 5% both for $\varphi_{RLP}$ and $\varphi_{max}$. Then the average of the closest three values was calculated using each of these parameters and presented in the final results.

### 2.3. Determination of Workability and Static Rheological Properties

Workability of the tested compositions was estimated at a concrete age $t_{age}$ of 10 min by means of the Haegermann flow table test in accordance with EN 1015-3 [35].

The static rheological properties were measured using the constant shear rate test and the single-batch approach, which were thoroughly described by the authors in [31] and [36]. In summary, the constant shear rate test includes determination of the peak values of torque at the lowest possible constant shear rate, or, more accurately, rotational velocity, at specific concrete ages. The peak values obtained for torque were used to calculate SYS values, while the development of SYS over time provides the structural build-up rate in the material. In the single-batch approach, measurements of peak torque at all ages are performed on a single sample. The alternative is the multi-batch approach, which uses a discrete sample for every concrete age or resting time under investigation in order to prevent continuous disturbance of material and to avoid possibly underestimated results for the structural build-up rate. The authors showed that for cement pastes and cement-based mortars of medium consistency, single-batch approach can be a solid alternative to the multi-batch approach due to minor differences in the results as obtained. Additionally, the time and material savings, the labour efficiency, and the better applicability for in situ testing of the single-batch approach are highlighted here.

A Viskomat XL Rheometer (Schleibinger Geräte Teubert und Greim GmbH, Germany) was used in this study. It is a dual-head, Couette-type rotational rheometer equipped with a ribbed cell with a height of 170 mm and an inner diameter of 135 mm and with a six-blade Vane probe; the height of the blades is 69 mm, the diameter 69 mm; the scheme is given in [37]. The sample volume is 3 L. The torque capacity ranges from 5 to 10^4^ N·mm, the torque resolution being 0.05 N·mm. Angular resolution is equal to 0.05°. The rotational velocity range is 0.0001 to 80 rpm. The device is applicable to concretes with a maximum particle size of not greater than 8 mm; particle sizes up to 16 mm may however also be admissible depending on the testing protocol and the shape of the aggregates. In this research, the applied constant rotational velocity was 0.3 rpm. Static yield stress measurements were conducted at $t_{age}$ of 20, 40, 60 and 80 min. No pre-shear was exerted. However, a zero static yield stress measurement was performed at $t_{age}$ of 10 min, which provided proper rotor positioning in the sample before the main tests [31]. To prevent excessive disturbance of the sample, each measurement was broken off as soon as the peak value of torque was reached; the corresponding technique is described in [36]. The temperature of the freshly prepared concrete was 20 ± 2 °C and was maintained at 20 ± 0.5 °C during the entire testing period by means of a temperature control module. The top of the cell was closed with a custom-designed plastic cover to prevent evaporation of water from the sample.

To calculate static yield stress $\tau_{0}$ from the measured values of torque T, the following equation was applied [38]:

(4) $$\tau_{0}=\frac{T}{2r^{2}\pi h}$$

where r and h are the radius and the height of the probe, respectively.

The structural build-up rate $A_{thix}$ was estimated using Roussel’s linear model [25] as it ensured a very good fit of the results obtained in this investigation; see Equation (5).

(5) $$\tau_{0}\left(t_{i}\right)=\tau_{0}\left(t_{0}\right)+A_{thix}\cdot t_{rest}$$

where $\tau_{0}\left(t_{0}\right)$ and $\tau_{0}\left(t_{i}\right)$ are the initial SYS and the SYS at $t_{rest}$, respectively, while $t_{rest}$ is resting time defined as $t_{rest}$ = $t_{age}-t_{0}$.

For each concrete composition, the measurement of the static properties was repeated twice, and in the cases when the difference between the values of the structural build-up rate exceeded 15%, a third measurement was performed. Then the average of the three values was calculated and presented as the final result.

## 3. Results and Discussion

### 3.1. Effect of the Volume Fraction

The effect of the volume fraction of aggregates on the structural build-up of concrete was studied under condition of constant surface area of aggregates per unit volume of paste, which was equal to 7.25 m^2^/L. The volume fraction φ was varied from 0.35 to 0.55 in increments of 0.05. For the given surface area, the volume fraction of 0.55 was the limit for testing; the mixtures having a higher aggregate content were too stiff.

**Figure 2:**
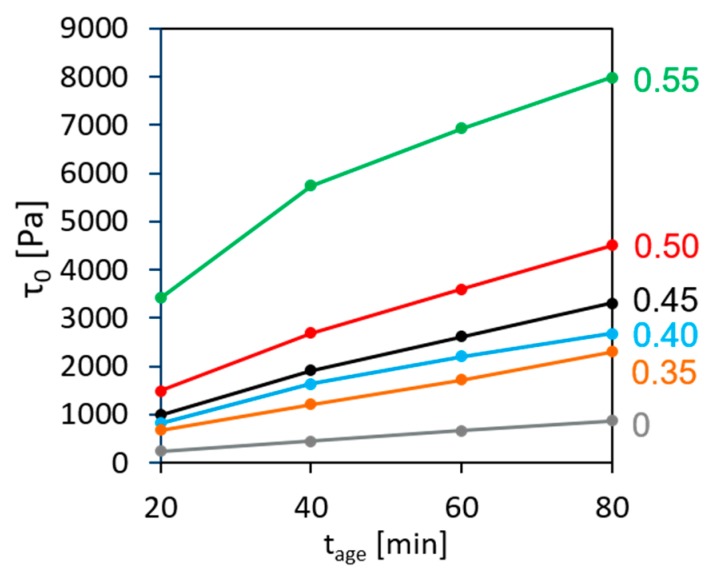
Development of static yield stress over age of concrete with various volume fractions of aggregates.

Figure 2 presents the evolution of static yield stress $\tau_{0}$ over the age of concrete $t_{age}$ (period of time elapsed after addition of water to the dry components) for the compositions under investigation.

The results show that in the present case the structural build-up during the first 80 min can be adequately described by Roussel’s linear model [25]. The values of the initial SYS for concrete ranged from 559 to 3743 Pa, increasing with the aggregate content while for the cement paste the initial SYS was merely 223 Pa. Structural build-up rate $A_{thix}$ increased correspondingly from 10.5 Pa/min for the cement paste to 25 and up to 98 Pa/min for concrete, again depending on the aggregate content.

**Figure 3:**
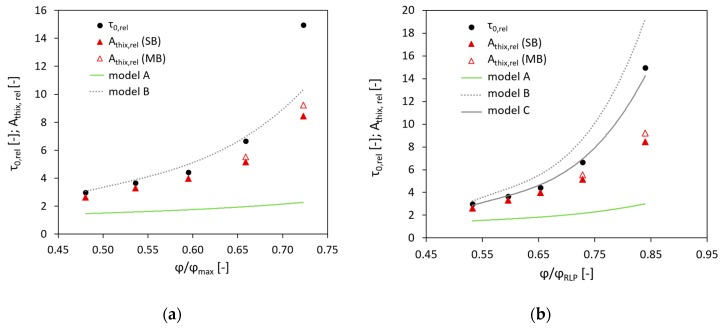
Relative initial SYS and relative structural build-up rate versus relative volume fraction calculated using (**a**) maximum volume fraction and (**b**) random loose packing fraction. Model A is Chateau–Ovarlez–Trung model, [η] = 2.5; model B is modified Chateau–Ovarlez–Trung model, [η] = 5.6; model C is modified Chateau–Ovarlez–Trung model, [η] = 5.1.

To compare the experimental results with the prediction provided by the Chateau–Ovarlez–Trung model and its modifications, relative values of the initial static yield stress $\tau_{0,\;rel}$ and the structural build-up rate $A_{thix,\;rel}$ were calculated and plotted versus the ratio between the volume fraction and the maximum volume fraction; see Figure 3a. Then similar calculations were performed using the values of the random loose packing fraction instead of the maximum volume fraction; see Figure 3b.

Firstly, it should be noted that the relative initial SYS and the relative $A_{thix}$ have comparable values only for the three lower volume fractions of aggregate (φ = 0.35, 0.40, 0.45). As the volume fraction increases to φ of 0.50 and 0.55, and the distance between aggregate particles decreases, the increase in relative $A_{thix}$ becomes less pronounced in comparison to that in the relative initial SYS. Primarily, the authors assumed that the reason for that was the measurement technique, since the single-batch approach (SB) was used. Multiple disturbance of a single sample can potentially have a more prominent effect on the obtained results when testing concrete with a high volume fraction of aggregate, especially if the mixture contains coarse aggregates. Both these features lead to a higher level of heterogeneity in the sheared region; hence, we repeated the measurements for the volume fractions of 0.50 and 0.55 using the multi-batch approach (MB). Although the structural build-up rate was found to be slightly higher (57 Pa/min instead of 53 Pa/min for φ = 0.50, and 95 Pa/min instead of 87 Pa/min for φ = 0.55), the previously observed trend did not change.

Since the models discussed further were originally designed to characterise the yield stress of the suspensions, they are to be firstly applied to predict the development of the initial SYS with increasing volume fraction of aggregates. Then the applicability of the models for predicting parameter $A_{thix}$ will be discussed.

As presented in Figure 3, the Chateau–Ovarlez–Trung model, i.e., model A, could not adequately describe the experimental data neither in the case using the values of $\varphi_{max}$, as in the initial form of the equation (Equation (6)), nor in the case of implementing the values of $\varphi_{RLP}$ instead of $\varphi_{max}$, as was done by Mahaut et al. [24], Lecompte et al. [26] and Perrot et al. [27].

(6) $$\tau_{0,rel.}=\sqrt{\left(1-\varphi \right){\left(1-\varphi /\varphi_{max}\right)}^{-2.5\varphi_{max}}}$$

Trying to achieve better agreement, the authors introduced the influence of the aggregate shape into the equation. For this purpose, the value of 2.5 in the exponent was substituted by the intrinsic viscosity of particles [η]. This parameter represents the individual particles’ effect on the viscosity of suspensions and depends both on the shape and on the volume fraction of suspended particles [39]. Intrinsic viscosity was considered in the Krieger-Dougherty Law [18], which was used as a basis for the Chateau–Ovarlez–Trung model. In the end both Krieger and Dougherty [18] and Chateau et al. [23] used [η] = 2.5 to describe the properties of the latexes and of the suspensions of spherical particles in yield-stress fluids, respectively. This value originates from Einstein’s theoretical prediction described in [17] and represents the intrinsic viscosity of ideal spherical particles in low-concentrated suspensions. However, since the shape of the aggregates used for concrete production normally deviates from that of an ideal sphere, and taking into consideration the research of Hafid et al. [12], who demonstrated therewith the effect of the particle shape on the relative yield stress of model-fluid suspensions, it was decided to modify the Chateau–Ovarlez–Trung model by increasing the value of [η].

Szecsy [40] suggested a nomogram presenting the relationship between the intrinsic viscosity and the circularity of aggregate particles, which was later used by Choi et al. [22] to improve a model for the prediction of concrete pumping behaviour. In the present research, the average circularity of the aggregate was equal to 0.85, which corresponds to [η] = 5.6 on Szecsy’s nomogram. The modified Chateau–Ovarlez–Trung model with this value of intrinsic viscosity is shown as model B in Figure 3. In the case of applying the values of $\varphi_{max}$, as in Figure 3a, this model provided sufficiently accurate agreement with the experimental results up to the volume fraction of 0.50. Beyond this threshold, the relative initial SYS was considerably underestimated. In the case of using the values of $\varphi_{RLP}$ (Figure 3b), the model overestimated the relative initial SYS.

Finally, the value of [η] was varied until the best fit of the experimental data was obtained, yielding with [η] = 5.1 for the random loose packing; see model C in Figure 3b. For the maximum packing concept, no [η] value was adequate to provide good agreement of the model and experimental data. It should be added that the intrinsic viscosity of 5.1 is valid for the particles with the sphericity of approximately 0.88.

Thus, in this research, the experimental data showing the relationship between the initial SYS and the volume fraction of aggregates can be described by Equation (7):

(7) $$\tau_{0,rel.}=\sqrt{\left(1-\varphi \right){\left(1-\varphi /\varphi_{RLP}\right)}^{-\left[\eta \right]\varphi_{RLP}}},$$

where [η] = 5.1.

It is worthwhile mentioning that the applied value of [η] is still close to the one obtained using Szecsy’s nomogram ([η] = 5.6), considering that many possible sources of error exist. Note that sphericity was determined on the particular samples and its average value was used. While the tested systems are polydisperse, their particle size distributions vary, making the average sphericity of each individual fraction different. In further research, the authors intend to assess the opportunity to optimizing Szecsy’s nomogram for highly polydisperse aggregate compositions in order to improve the accuracy of predicting the intrinsic viscosity.

**Figure 4:**
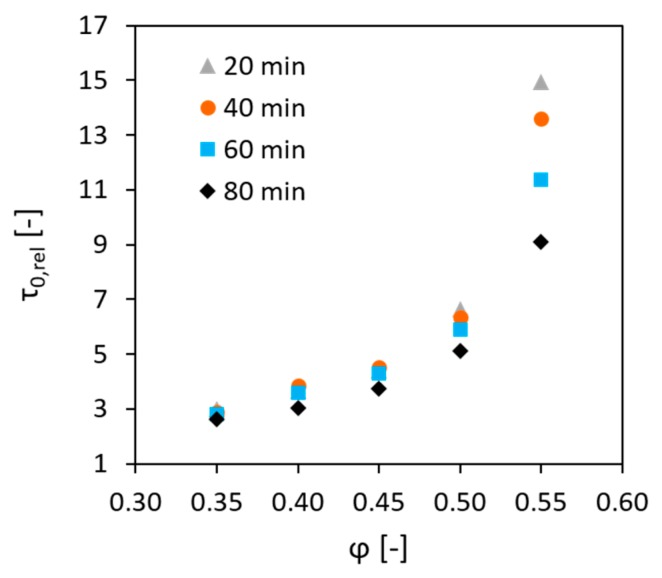
Time-dependency of the development of relative static yield stress with increase in the volume fraction of aggregates in concrete.

Furthermore, it should be explained why the modified equation of Mahaut et al. [24], $\;A_{thix,\;rel}=f\left(\varphi \right)=\sqrt{\left(1-\varphi \right)\;{\left(1-\varphi /\varphi_{RLP}\right)}^{-\left[\eta \right]\varphi_{RLP}}}$, cannot provide a fit for the experimental result in this investigation. In their research, the authors relied upon the Roussel’s model $\tau_{0}\left(t_{i}\right)=\tau_{0}\left(t_{0}\right)+A_{thix}\cdot t_{rest}$ and assumed that the effect of aggregates on the yield stress described by the expression f(φ) is time-independent. This assumption seems logical, because φ and $\varphi_{RLP}$ are not subjected to change after the concrete mixture is prepared. Mahaut’s model for the relative $A_{thix}$ was proved to be valid for the short resting times and in the case when concrete was stirred before each SYS measurement. However, in the case of longer resting times and static conditions, as encountered in this research, the evolution of the relative SYS with the increasing volume fraction changed over time; see Figure 4.

Moreover, it was observed that the extent of this change depends the volume fraction of the aggregates. Being almost negligible at φ = 0.35, it became very prominent at φ = 0.55. This behaviour can occur for various reasons. The first possible reason is that the properties of the actual constitutive paste in concrete may differ from those of the cement paste, which was prepared separately, and may be dependent on the aggregate content and composition. As discussed in the introduction, higher volume fraction of aggregates, especially of finer ones, should result in higher content of water required for wetting their surfaces; hence, the actual w/b in the constitutive paste is reduced. Additionally, aggregates act as grinding bodies during mixing, leading to better dispersion of cement in the mixture, thus making it more reactive. The second reason is that the finer aggregate particles can possibly affect the kinetics of the structuration processes by acting as a surface for precipitation of the early hydration products. However, we have no experimental evidence at this stage proving any of these hypotheses. They will be the subject of follow-up research. Deeper understanding of the mechanism lying under the time-dependent effects of aggregates on the structural build-up of concrete should ensure the further development of the model for the accurate prediction of $A_{thix}$.

### 3.2. Effect of the Surface Area

The second stage of the research was dedicated to the effect of the surface area of aggregates per unit volume of cement paste $A_{ag}^{V_{p}}$ on the structural build-up of concrete. Three volume fractions were selected, φ = 0.35, 0.45, and 0.55, for which $A_{ag}^{V_{p}}$ was varied as follows: 5.00, 7.25, 10.00 m^2^/L.

**Figure 5:**
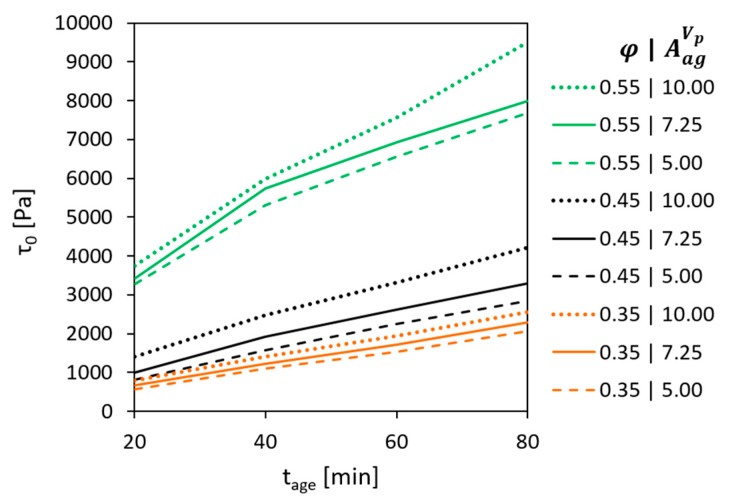
Development of static yield stress over age of concrete: effect of the volume fraction and the surface area of aggregates per unit volume of cement paste.

**Figure 6:**
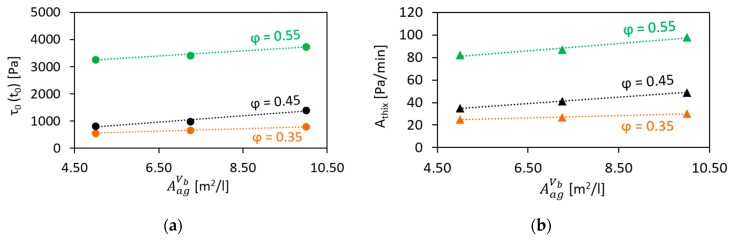
Effect of the surface area of aggregates per unit volume of cement paste on (**a**) the initial SYS and (**b**) the structural build-up rate of concrete.

The development of static yield stress $\tau_{0}$ with increasing age of concrete $t_{age}$ for the compositions studied is depicted in Figure 5, while Figure 6 presents the variations in the initial SYS and in the structural build-up rate with increasing surface area of the aggregates per unit volume of cement paste.

The results obtained showed that within the investigated range, growth in $A_{ag}^{V_{p}}$ leads to a linear increase in both initial SYS and $A_{thix}$ for all tested volume fractions. Moreover, the described effect becomes generally more pronounced at higher volume fractions.

**Figure 7:**
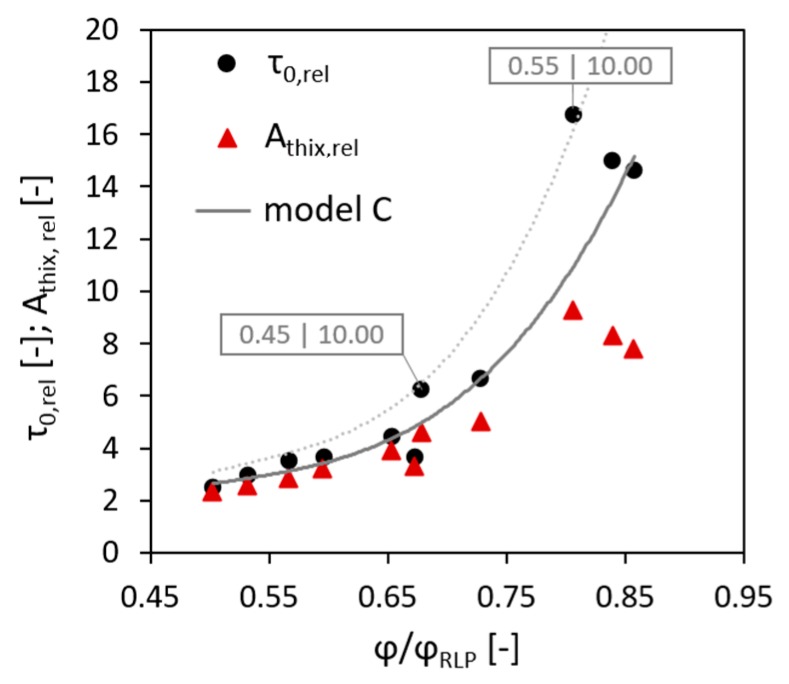
Relative initial SYS and relative structural build-up rate versus relative volume fraction: the effect of surface area of aggregates per unit volume of cement paste. Model C is modified Chateau–Ovarlez–Trung model, [η] = 5.1.

It should be noted that adding the results achieved in this chapter to the plot in Figure 3a leads to higher fluctuation of the data points and their noticeable deviation from the model in the case of $A_{ag}^{V_{p}}$ = 10.00 for the volume fractions of 0.45 and 0.55; see Figure 7.

This deviation may be caused by considerably higher contents of fine sand in the respective compositions, which in turn resulted in the enhanced structuration of the concrete mixture. Thus, the behaviour as observed may count in favour of the possibility of physical-chemical interactions between cement paste and aggregate particles. In terms of the model, the higher volume fraction of fine particles can be taken into account by increasing the intrinsic viscosity parameter, which, according to [39], is higher in concentrated suspensions due to crowding. The dotted line presented in Figure 6 was obtained with [η] = 5.6.

**Figure 8:**
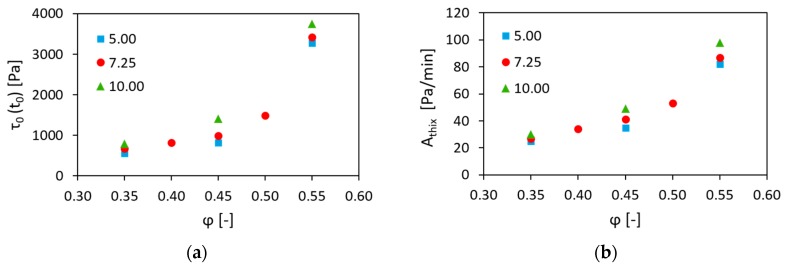
Combined effect of the volume fraction and the surface area of aggregates per unit volume of cement paste on (**a**) the initial SYS and (**b**) the structural build-up rate of concrete.

The practical importance of the experimental results obtained consists in the possibility of fine tuning the static rheological properties by varying the surface area of aggregates per unit volume of cement paste; see Figure 8.

For example, concrete compositions with φ = 0.45, $A_{ag}^{V_{p}}$ = 10.00 m^2^/L and with φ = 0.50, $A_{ag}^{V_{p}}$ = 7.25 m^2^/L were characterized by the similar initial SYS (1405 Pa and 1490 Pa, respectively) and the comparable structural build-up rates, i.e., 49 Pa/min and 53 Pa/min, while having different workability, i.e., spread diameter of 200 mm and 188 mm, respectively; see Table 2. Thus, varying both φ and $A_{ag}^{V_{p}}$ parameters can provide more freedom in terms of the mix design, allowing the production of concrete compositions with the specified rheological behaviour.

## 4. Conclusion and Outlook

The effects of the volume fraction of aggregates and the surface area of aggregates per unit volume of cement paste on the two main parameters defining the static rheological properties of concrete, i.e., initial SYS and structural build-up rate, were evaluated and discussed.

A modification of the Chateau–Ovarlez–Trung model was proposed, which was able to provide a sufficiently accurate mathematical description of the relationship between the relative initial SYS and the relative volume fraction for the investigated concrete compositions with varied volume fraction and constant surface area of aggregates per unit volume of cement paste. To obtain that, the values of the maximum volume fraction were substituted by the random loose packing fraction, as previously proposed by the other researchers. The value of intrinsic viscosity was increased from 2.5 to 5.1.

It was found that in the case when the structural build-up rate of concrete is determined in the static conditions, the same expression cannot be successfully applied in predicting the relative $A_{thix}$, because the evolution of the relative SYS with increasing volume fraction is time-dependent. Moreover, the time-dependency becomes more pronounced with higher aggregate content in the concrete.

Increase in the surface area of the aggregates per unit volume of cement paste resulted in linear growth in both the initial SYS and the structural build-up rate.

From a practical point of view the results achieved have shown that the static rheological properties of concrete can be significantly affected by variation of the volume fraction of aggregates and thereafter fine-tuned by changing the surface area of aggregates or, in other words, by modifying the particle size distribution.

From a scientific point of view, it is important to analyse further how the state of the cement paste in concrete is influenced by the aggregate composition, especially by the fine fractions of sand. The results of this research enable the posing of a hypothesis that fine sand particles may have physical-chemical interactions with the cement paste, leading to enhanced structuration in concrete. This assumption will be the subject of follow-up research, which should also result in finding a proper model to describe the effect of aggregates on the structural build-up rate of concrete.

## Figures and Tables

**Table 1:** Grading curves of the aggregate compositions.

φ [-]	0.35			0.40	0.45			0.50	0.55		
$A_{ag}^{V_{p}}$ [m^2^/L]	5.00	7.25	10.00	7.25	5.00	7.25	10.00	7.25	5.00	7.25	10.00
**Sieve Size [mm]**	**Percentage Passing [%]**										
0.125	0	0	0	0	0	0	0	0	0	0	0
0.25	12.0	21.5	34.0	15.4	5.0	10.6	18.5	8.0	1.5	4.0	9.0
0.5	25.5	41.0	62.0	31.5	13.0	24.5	36.5	18.3	4.5	13.0	22.3
1	41.5	58.4	76.0	48.0	27.3	40.0	53.5	31.6	18.0	28.5	36.9
2	58.0	74.0	86.0	65.0	49.0	57.5	69.5	48.9	40.0	47.5	54.5
4	78.0	87.5	94.0	82.2	73.5	76.5	85.0	71.2	68.0	71.0	75.5
8	100.0	100.0	100.0	100.0	100.0	100.0	100.0	100.0	100.0	100.0	100.0
**Parameter**	**Value**										
$\varphi_{RLP}$ [-]	0.70	0.66	0.62	0.67	0.67	0.69	0.66	0.69	0.64	0.66	0.68
$\varphi_{max}$ [-]	0.76	0.73	0.68	0.75	0.75	0.76	0.74	0.76	0.73	0.76	0.76
$\varphi /\varphi_{RLP}$ [-]	0.50	0.53	0.57	0.60	0.67	0.65	0.68	0.73	0.86	0.84	0.81
$\varphi /\varphi_{max}$ [-]	0.46	0.48	0.52	0.54	0.60	0.59	0.61	0.66	0.75	0.72	0.72

**Table 2:** Concrete compositions under investigation.

φ [-]	0.35			0.40	0.45			0.50	0.55			Cement paste
$A_{ag}^{V_{p}}$ [m^2^/L]	5.00	7.25	10.00	7.25	5.00	7.25	10.00	7.25	5.00	7.25	10.00	
**Component**	**Amount per 4 L of Concrete [kg]**											
CEM I 42.5 R	3.598	3.598	3.598	3.321	3.045	3.045	3.045	2.768	2.491	2.491	2.491	5.536
Sand fr. 0.125–0.250 mm	0.445	0.798	1.261	0.653	0.239	0.506	0.882	0.423	0.087	0.233	0.525	-
Sand fr. 0.25–0.50 mm	0.501	0.723	1.039	0.683	0.382	0.663	0.859	0.547	0.175	0.525	0.775	-
Sand fr. 0.5–1.0 mm	0.594	0.646	0.519	0.700	0.682	0.739	0.811	0.707	0.787	0.904	0.851	-
Sand fr. 1–2 mm	0.612	0.579	0.371	0.721	1.035	0.835	0.763	0.914	1.283	1.108	1.026	-
Sand fr. 2–4 mm	0.742	0.501	0.297	0.729	1.169	0.906	0.739	1.181	1.632	1.370	1.224	-
Gravel fr. 4–8 mm	0.816	0.464	0.223	0.755	1.264	1.121	0.716	1.527	1.866	1.691	1.428	-
Water	1.439	1.439	1.439	1.329	1.218	1.218	1.218	1.107	0.996	0.996	0.996	2.216
**Parameter**	**Value**											
Spread diameter [mm]	220	210	210	210	210	210	200	188	177	168	166	235

## Terms and Definitions

**Volume fraction**	Volume concentration of particles in the unit volume of material: e.g., suspension, concrete
**Maximum volume fraction**	Ratio of the true volume of particles to the volume occupied by them in a maximally compacted state
**Random loose packing fraction**	Ratio of the true volume of particles to the volume occupied by them in a random loose state, i.e., without compaction
**Surface area of aggregate per unit volume of cement paste**	Total area of surfaces of all aggregate particles distributed in the unit volume of cement paste
**Sphericity**	Ratio of the surface area of a sphere to the surface area of an actual, irregularly shaped particle of equivalent volume
**Intrinsic viscosity**	Measure of the effect of individual particles on the viscosity of suspensions; dependent on particle shape and concentration
**Static yield stress**	Stress which is required to initiate flow
**Structural build-up**	Evolution of rheological properties of a cementitious material over time due to flocculation and early hydration
**Structural build-up rate**	Rate of increase in the static yield stress of a cementitious material over time
**Relative static yield stress**	Ratio of the static yield stress of suspension, e.g., concrete, to the static yield stress of suspending medium, e.g., cement paste
**Relative structural build-up rate**	Ratio of the structural build-up rate of suspension, e.g., concrete, to the structural build-up rate of suspending medium, e.g., cement paste
**Constant shear rate test**	Rheometry method for measuring static yield stress; a low, constant shear rate is applied until shear stress reaches its peak or plateau value
**Single-batch approach**	A technique used to determine the evolution of static yield stress over time, in which the measurements of peak torque at all ages are performed on a single sample of material
**Multi-batch approach**	A technique used to determine the evolution of the static yield stress over time, in which measurement of peak torque at each particular age is performed on a discrete, individually prepared sample of the same material

## References

[1] Wangler T., Lloret E., Reiter L., Hack N., Gramazio F., Kohler M., Bernhard M., Dillenburger B., Buchli J., Roussel N. (2016). Digital Concrete: Opportunities and Challenges. RILEM Tech. Lett..

[2] Roussel N. (2018). Rheological requirements for printable concretes. Cem. Concr. Res..

[3] Reiter L., Wangler T., Roussel N., Flatt R.J. (2018). The role of early age structural build-up in digital fabrication with concrete. Cem. Concr. Res..

[4] Omran A.F., Khayat K.H. (2012). Effect of SCC Mixture Composition on Thixotropy and Formwork Pressure Effect of SCC Mixture Composition on Thixotropy and Formwork Pressure. J. Mater. Civ. Eng..

[5] Ovarlez G., Roussel N. (2006). A physical model for the prediction of lateral stress exerted by self-compacting concrete on formwork, Mater. Struct. Constr..

[6] Billberg P.H., Roussel N., Amziane S., Beitzel M., Charitou G., Freund B., Gardner J.N., Grampeix G., Graubner C.A., Keller L. (2014). Field validation of models for predicting lateral form pressure exerted by SCC. Cem. Concr. Compos..

[7] Tokhmechi B., Velayati A., Kazemzadeh E., Soltanian H. (2015). Gas migration through cement slurries analysis: A comparative laboratory study. Int. J. Min. Geo-Eng..

[8] Lloret E., Shahab A.R., Linus M., Flatt R.J., Gramazio F., Kohler M., Langenberg S. (2015). Complex concrete structures: Merging existing casting techniques with digital fabrication. Comput. Des..

[9] Buswell R.A., Leal de Silva W.R., Jones S.Z., Dirrenberger J. (2018). 3D printing using concrete extrusion: A roadmap for research. Cem. Concr. Res..

[10] Nerella V.N., Hempel S., Mechtcherine V. (2019). Effects of layer-interface properties on mechanical performance of concrete elements produced by extrusion-based 3D-printing. Constr. Build. Mater..

[11] Van Damme H. (2018). Concrete material science: Past, present, and future innovations. Cem. Concr. Res..

[12] Hafid H., Ovarlez G., Toussaint F., Jezequel P.H., Roussel N. (2016). Effect of particle morphological parameters on sand grains packing properties and rheology of model mortars. Cem. Concr. Res..

[13] Mehdipour I., Khayat K.H. (2018). Understanding the role of particle packing characteristics in rheo-physical properties of cementitious suspensions: A literature review. Constr. Build. Mater..

[14] Fuller W.B., Thompson S.E. (1907). The laws of proportioning concrete. Trans. ASCE.

[15] Andreasen A.H.M. (1930). Ueber die Beziehung zwischen Kornabstufung und Zwischenraum in Produkten aus losen Körnern (mit einigen Experimenten). Kolloid-Zeitschrift.

[16] Funk J.E., Dinger D. (1994). Predictive Control of Crowded Particulate Suspension Applied to Ceramic Manufacturing.

[17] Einstein A. (1905). Eine neue Bestimmung der Moleküldimensionen. Ann. Phys..

[18] Krieger I.M., Dougherty T.J. (1959). A Mechanism for Non-Newtonian Flow in Suspensions of Rigid Spheres. Trans. Soc. Rheol..

[19] Chong J.S., Christiansen E.B., Baer A.D. (1971). Rheology of concentrated suspensions. J. Appl. Polym. Sci..

[20] Quemada D. (1977). Rheology of concentrated disperse systems and minimum energy dissipation principle—I. Viscosity-concentration relationship. Rheol. Acta.

[21] Farris R.J. (1968). Prediction of the Viscosity of Multimodal Suspensions from Unimodal Viscosity Data. Trans. Soc. Rheol..

[22] Choi M.S., Kim Y.J., Kim J.K. (2014). Prediction of Concrete Pumping Using Various Rheological Models. Int. J. Concr. Struct. Mater..

[23] Chateau X., Ovarlez G. (2008). Homogenization approach to the behavior of suspensions of noncolloidal particles in yield stress fluids. J. Rheol..

[24] Mahaut F., Mokéddem S., Chateau X., Roussel N., Ovarlez G. (2008). Effect of coarse particle volume fraction on the yield stress and thixotropy of cementitious materials. Cem. Concr. Res..

[25] Roussel N. (2006). A thixotropy model for fresh fluid concretes: Theory, validation and applications. Cem. Concr. Res..

[26] Lecompte T., Perrot A., Picandet V., Bellegou H., Amziane S. (2012). Cement-based mixes: Shearing properties and pore pressure. Cem. Concr. Res..

[27] Perrot A., Lecompte T., Estellé P., Amziane S. (2013). Structural build-up of rigid fiber reinforced cement-based materials. Mater. Struct. Constr..

[28] Le T.T., Austin S.A., Lim S., Buswell R.A., Gibb A.G.F., Thorpe T. (2012). Mix design and fresh properties for high-performance printing concrete. Mater. Struct..

[29] Lindemann H., Gerbers R., Ibrahim S., Dietrich F., Herrmann E., Dröder K., Raatz A., Kloft H., Wangler T., Flatt R.J. (2019). Development of a Shotcrete 3D-Printing (SC3DP) Technology for Additive Manufacturing of Reinforced Freeform Concrete Structures. RILEM Bookseries.

[30] Lowke D., Bittnar Z., Bartos P.J.M., Němeček J., Šmilauer V., Zeman J. (2009). Interparticle Forces and Rheology of Cement Based Suspensions. Nanotechnology in Construction 3.

[31] Ivanova I., Mechtcherine V. (2020). Evaluation of Structural Build-Up Rate of Cementitious Materials by Means of Constant Shear Rate Test: Parameter Study. Rheology and Processing of Construction Materials.

[32] Stark J., Wicht B. (2000). Zement und Kalk: Der Baustoff als Werkstoff, F.A.

[33] Wadell H. (1933). Sphericity and Roundness of Rock Particles. J. Geol..

[34] De Larrard F. (1999). Concrete Mixture Proportioning.

[35] German Institute for Standardisation (Deutsches Institut für Normung) (2007). Methods of Test for Mortar for Masonry—Part 3: Determination of Consistence of Fresh Mortar (by Flow Table).

[36] Ivanova I., Mechtcherine V. (2020). Possibilities and challenges of constant shear rate test for evaluation of structural build-up rate of cementitious materials. Cem. Concr. Res..

[37] Eslami Pirharati M., Ivanov D., Krauss H.-W., Schilde C., Lowke D. (2020). Rheology and Processing of Composite Materials. RILEM Bookseries.

[38] Heirman G., Hendrickx R., Vandewalle L., Van Gemert D., Feys D., De Schutter G., Desmet B., Vantomme J. (2009). Integration approach of the Couette inverse problem of powder type self-compacting concrete in a wide-gap concentric cylinder rheometer. Part II. Influence of mineral additions and chemical admixtures on the shear thickening flow behavior. Cem. Concr. Res..

[39] Struble L., Sun G.K. (1995). Viscosity of Portland cement paste as a function of concentration. Adv. Cem. Based Mater..

[40] Szecsy R.S. (1997). Concrete Rheology. Ph.D. Thesis.

